# Design and Development of a Disposable Superfine Catheter for Visual Examination of Bile Ducts and Related Animal Experiments

**DOI:** 10.3389/fsurg.2022.877040

**Published:** 2022-05-02

**Authors:** Jin-Yong Hao, Yao-Ping Zhang, Xiao-Jun Huang

**Affiliations:** ^1^ Department of Gastroenterology, Lanzhou University Second Hospital, Lanzhou, Gansu, China; ^2^ Gansu Provincial Digestive Endoscopy Engineering Research Center, Lanzhou, Gansu, China

**Keywords:** biliary disease, per-oral choledochoscope, visual, disposable, superfine

## Abstract

**Objective:**

To design and develop a disposable superfine catheter system for visual examination of bile and pancreatic ducts and predict its clinical application value.

**Methods:**

The superfine triple-lumen catheter and miniature photography technology were used to design and produce a disposable superfine catheter for visual examination of bile and pancreatic ducts, and animal experiments were conducted to compare said catheter and SpyGlass™.

**Results:**

The designed and developed disposable superfine catheter for visual examination of bile ducts with a diameter of 2.4 mm could enter the third-order and fourth-order bile ducts in the animal liver and also the gallbladder via the cystic duct for observation. The said catheter has a water injection rate of 0.8 mL/s, 0.16 megapixels, a resolution of 400 × 400, a depth of field of 0.3 to 20 mm, and a tilting up angle >90°.

**Conclusion:**

The new disposable catheter for visual examination of bile ducts has a superfine diameter, easier operation, and clearer imaging, and is expected to have a higher clinical practical value.

## Background

The hepatobiliary system is an important part of the human digestive system comprising the liver, gallbladder, and a complex network of intrahepatic and extrahepatic bile ducts. These bile ducts, or channels, gather to form a vast three-dimensional structure called the biliary tree (1). According to the anatomical positions, the biliary system is divided into intrahepatic and extrahepatic bile ducts, the common hepatic duct, the cystic duct, and the gallbladder. Common biliary diseases include cholangitis, parasites, cholelithiasis, and bile duct malignancies.

According to statistics, about 20 million people suffer from cholelithiasis in the United States; the overall incidence of gallstone disease is 18.8% in Europe, with 9.5% occurring in males (2). Choledocholithiasis is very common in Asia. Literature shows that the proportion of patients with choledocholithiasis who have clinical symptoms is about 20% (3).

Cholangiocarcinoma (CCA) is the second most common primary hepatic malignancy after hepatocellular carcinoma (HCC), comprising about 15% of all primary liver tumors (4). The slow and silently evolving progression of CCA creates great difficulty for clinical treatment (5). Most of the poor prognoses of CCA result from a late diagnosis, leading to high clinical mortality rate and low postoperative survival. According to research statistics, the five-year survival rate after resection of bile duct malignancies is usually less than 40% and is less than 5% in patients with unresectable bile duct malignancies (6).

The current clinical diagnosis of CCA primarily relies on a combination of multiple imaging techniques, including ultrasound, computed tomography (CT), positron emission computed tomography (PET), and magnetic resonance imaging (MRI). Bile duct pathological tissue is mainly obtained through endoscopic retrograde cholangiopancreatography (ERCP) brush cytology or biopsy, however, bile duct brushing has a low positive rate and easily causes contamination. Bile duct biopsies present problems such as difficulty with biopsy site localization under X-ray and failure to make a section of the sample obtained by the excessively small per-oral choledochoscope biopsy forceps, leading to the difficulty of diagnosing bile duct malignancies (7).

Bakes designed a laryngoscope-like endoscope in 1923, which, for the first time, enabled direct visual observation of the common bile duct (8).

Researchers developed two different types of per-oral cholangioscopy (POCS) techniques in the late 1970s, i.e., the indirect POCS technique proposed by Kawai (9) et al. and the direct POCS technique proposed by Urakami (10) et al. Boston Scientific introduced a single-operator catheter-based POCS technique called SpyGlass™ in 2005 (11). However, according to the related literature, SpyGlass™ presents problems such as a thick diameter, inconvenient operation, low image pixels, a fragile optical fiber, vulnerable steerable wire, and expensive cost (12, 13).

Therefore, we intended to develop and produce a new disposable superfine catheter system for visual examination of bile and pancreatic ducts, which could enter bile and pancreatic ducts for visual observation through catheter insertion during ERCP and whose overall diameter would be minimized on the premise of not affecting the visual observation. The system would be designed to enter the intrahepatic third-order and fourth-order bile ducts and the cystic duct to achieve non-invasive visual observation of intrahepatic bile ducts and the gallbladder.

## Methods

A disposable superfine catheter system for visual examination of bile and pancreatic ducts was designed and compared with SpyGlass™ in terms of hardware performance and in animal experiments.

The catheter system consists of four parts, i.e., the front-end camera part, the examination tube part, the tail-end operation part, and the power supply and data output part. The catheter's front end consists of a miniature camera module and a lighting source. The tube body is in a triple-lumen structure and the tail end is a three-way operating handle.

**Figure 1:**
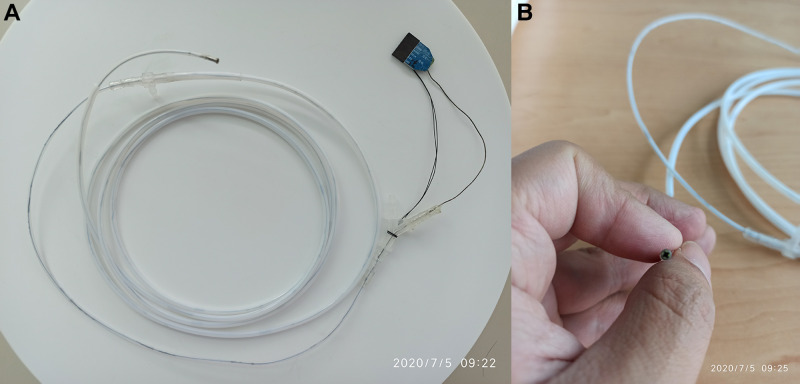
Disposable superfine catheter for visual examination of bile and pancreatic ducts. (**A**) Overall Catheter, (**B**) Catheter Front End.

Description of the specific design and production of the disposable superfine catheter system for visual examination of bile and pancreatic ducts is as follows: the camera part consists of a camera module surrounded by four LED beads and overall constitutes the cylindrical head end, with a diameter of 2.38 mm ± 0.05 mm and a length of 5.0 mm ± 0.03 mm. The LED beads are connected in series to the main control circuit, with the brightness adjustable through the adjustment of resistance. The camera resolution is 160 k (400 × 400), covering a 120° wide-angle range lighted by the front LED beads. The main body is a triple-lumen Teflon catheter, with a total length of 1.8–2.0 m and a circumference of about 2.4 mm, which is molded in one step. The three lumens consist of a superfine guidewire lumen with a diameter of 1.0 mm, allowing the passage of a 0.038-inch yellow zebra guidewire, a data cable transmission channel, with a diameter of about 0.5 mm, and a water injection/suction lumen that can simultaneously operate water injection, air injection, and negative pressure suction (refer to **Table 1** for the specific parameters). The tail end is a three-way connector structure, with a water/air inlet/outlet, a superfine guidewire inlet in the middle, and a cable and device inlet. A rubber ring is provided externally, which can tightly wrap around the imaging or photography catheter when said catheter is inserted via the device inlet, forming a sealed lumen that prevents water and air backflow (**Figure 1**).

**Table 1:** Parameters of disposable superfine catheter for visual examination of bile and pancreatic ducts.

Parameters	
Outer diameter	≤2.4 mm
Pixels	16*10^4^
Resolution	400 × 400
Angle of view (diagonal)	120°
Depth of field	3–200 mm
Scanning mode	Progressive scanning
Front-end length	5 mm (Inflexible part)

Comparison of hardware performance between the disposable superfine catheter for visual examination of bile and pancreatic ducts and SpyGlass™ DS.

**Figure 2:**
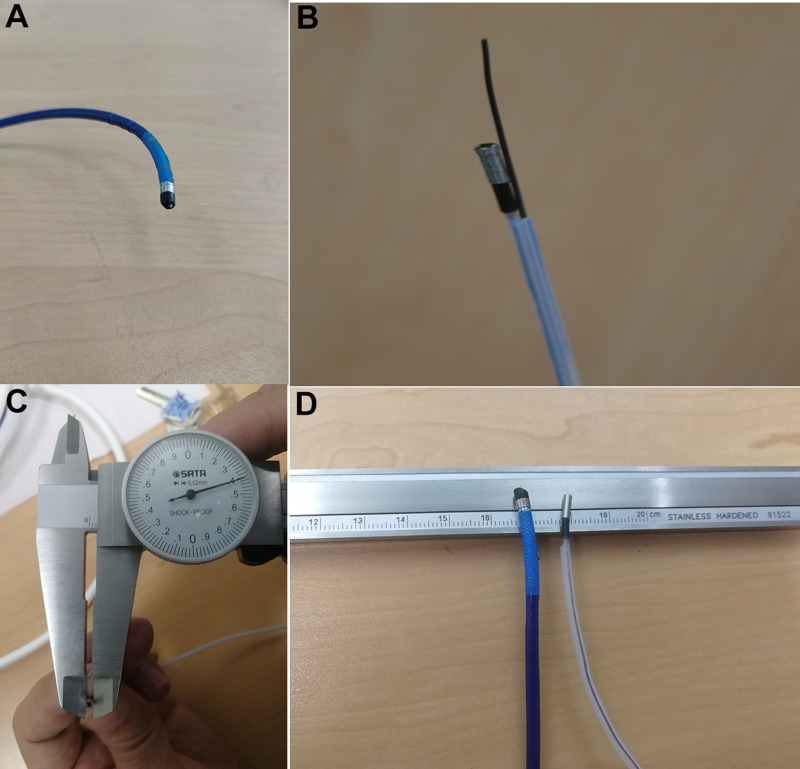
Comparison of front-end diameters. (**A**) Front End of SpyGlass™ DS, (**B**) Front End of Disposable Superfine Catheter for Visual Examination of Bile and Pancreatic Ducts, (**C**) Front-end Diameter of Disposable Superfine Catheter for Visual Examination of Bile and Pancreatic Ducts Measured with Micrometer, (**D**) Comparison of Front Ends of the Two.

Comparison of front-end diameters is shown in **Figure 2**. The measuring instrument was a micrometer, and the part measured was the camera end (2 mm from the top).

**Figure 3:**
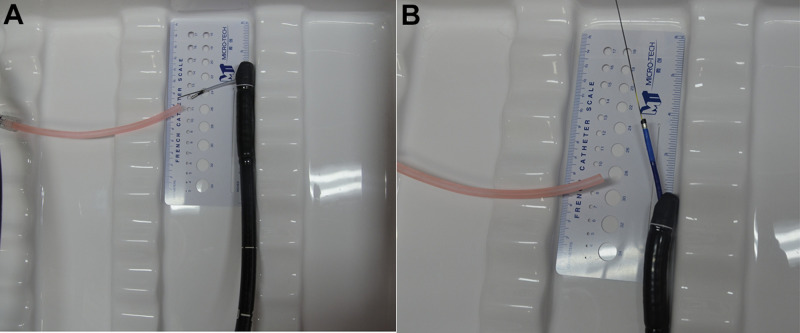
Comparison of front-end tilting up angles. (**A**) Disposable Superfine Catheter for Visual Examination of Bile and Pancreatic Ducts, (**B**) SpyGlass™ DS.

Comparison of front-end bendability is shown in **Figure 3**. The two different products were inserted in the duodenum forceps channel, with the front end extending 2.0 cm from the forceps channel outlet, and the duodenoscope forceps lifter was lifted to the maximum angle, so as to measure the front-end bending angle. The measuring instruments included a ruler and a protractor.

### Comparison of Water Injection Performance

**Figure 4:**
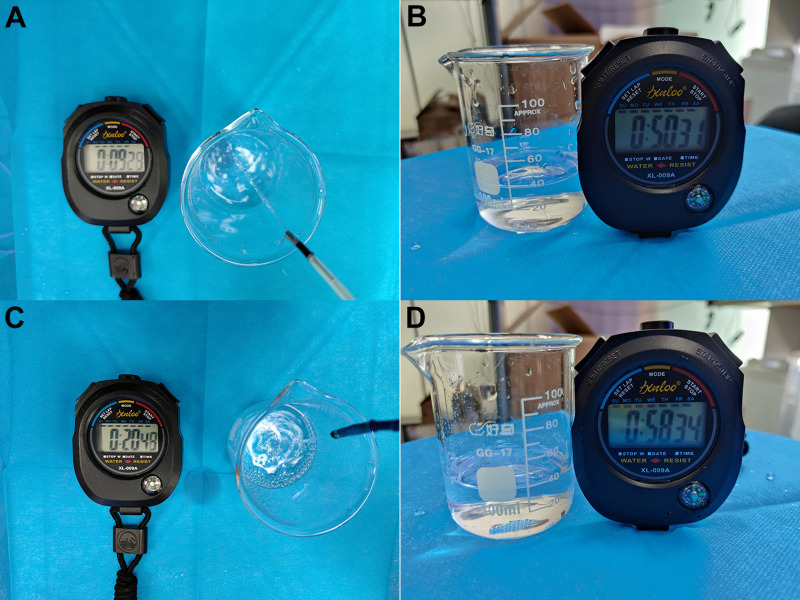
Comparison of water injection rates. (**A**) Water Injection by Disposable Superfine Catheter for Visual Examination of Bile and Pancreatic Ducts, (**B**) Time of 40 mL Water Injection by Disposable Superfine Catheter for Visual Examination of Bile and Pancreatic Ducts, (**C**) Water Injection by SpyGlass™ DS, (**D**) Time of 40 mL Water Injection by SpyGlass™ DS.

The water injection by a water pump in ERCP was simulated to compare the water injection rate (**Figure 4**). Bubble-free water was used in the experiments, and the water pump was the SpyGlass™ Irrigation Pump that supports SpyGlass™ DS.

After the pump was pressurized (adjusted to the maximum pressure), the time required to inject 40 mL of water into the measuring glass was observed separately for the two products. (Note: The total amount of 40 mL was adopted because repeated observation showed that the pump pressure would change after the pump continued to inject 40 mL of water.) The comparison observation experiment of water injection rates showed that the time required to inject 40 mL of water was 50.31 seconds for the disposal ERCP and photography catheter system (the prototype) and 58.34 s for SpyGlass™ DS.

### Animal Experiments

The animal experiments were approved by the Animal Experimentation Ethics Committee of Lanzhou University Second Hospital.

**Figure 5:**
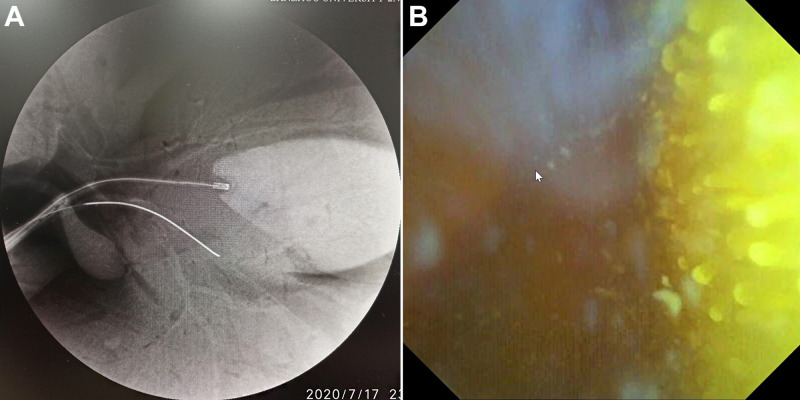
Catheter Entering Gallbladder Under X-ray (**A**) and Villi inside Gallbladder (**B**).

**Figure 6:**
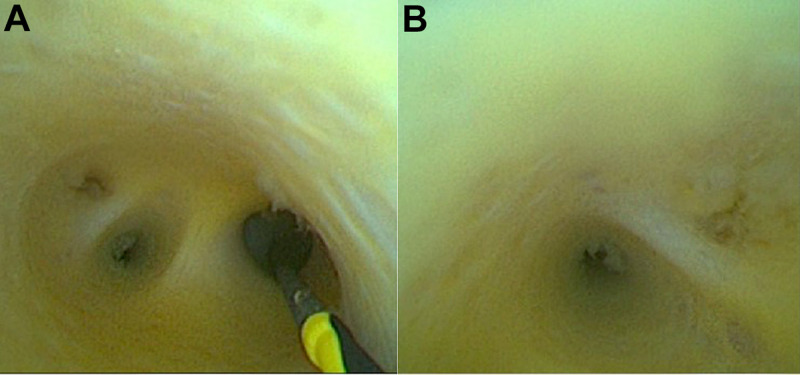
Porta Hepatis Bile Duct (**A**) and Intrahepatic Fourth-Order Bile Duct (**B**).

**Figure 7:**
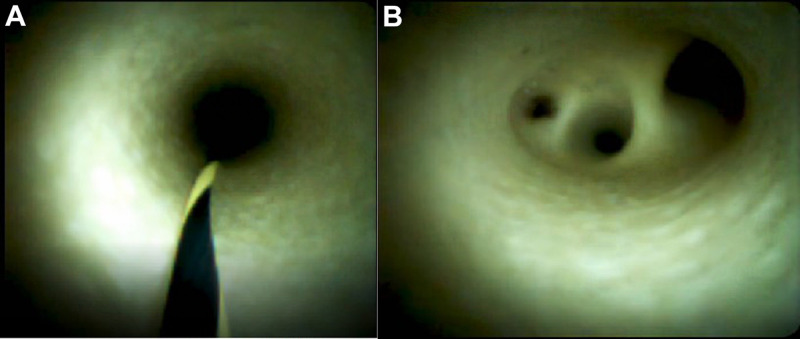
Bile Duct (**A**) and Porta Hepatis Bile Duct (**B**) Observed with SpyGlass™.

The duodenum of a miniature pig was dissected to find the duodenal papilla, and the disposable superfine catheter for visual examination of bile and pancreatic ducts was guided into the bile duct by the superfine guidewire and then advanced for observation. After entering the bile duct, the said catheter traveled upward for about 2 to 3 cm, then an independent opening was seen in the left wall of the common bile duct, which was nearly vertical and considered to be the cystic duct opening; the photography catheter and the guidewire were then withdrawn. The guidewire direction was adjusted to enter the gallbladder, and the catheter followed the guidewire into the gallbladder. The shapes of the guidewire and the photography catheter in the gallbladder were observed under X-ray (refer to **Figure 5A**). Bile was sucked from the water injection lumen of the photography catheter and normal saline was injected into said lumen for rinsing, after which, regular arrangements of fine and dense villi in the inner wall of the gallbladder were observed (refer to **Figure 5B**). After observation of the gallbladder, the guidewire and the photography catheter were withdrawn to the common bile duct to continue traveling upward through the bile duct for observation, and normal saline was used for rinsing intermittently in the observation process to keep the view clean and the bile duct filled. After traveling upward about 5 to 7 cm, the bile duct was seen to bifurcate into the left and right intrahepatic bile ducts. The guidewire direction was adjusted to enter the intrahepatic bile duct on one side and the photography catheter easily followed the guidewire into the intrahepatic bile duct. After entry, the photography catheter could continue to advance in the liver for visualizing the branch (third-order and fourth-order) bile ducts (refer to **Figure 6**). The same method was adopted for SpyGlass's entry into the miniature pig's bile duct for observation, and the images were kept for comparison (refer to **Figure 7**).

## Results

We successfully designed, developed, and produced a disposable superfine catheter system for visual examination of bile and pancreatic ducts. The comparison of the hardware performance and the observation of application in animal bile ducts showed the following advantages of the disposable superfine catheter system for visual examination of bile and pancreatic ducts: 1. superfine catheter diameter that allowed the catheter to enter the intrahepatic third-order and fourth-order bile ducts and the gallbladder; 2. efficient water injection; 3. high image resolution; 4. convenient operation (refer to **Table 2**).

**Table 2:** Performance comparison.

Name	Disposable superfine catheter system for visual examination of bile and pancreatic ducts	SpyGlass™ DS
Technical performance		
Number of surgeons	One	One
Operation	Simple and flexible	Complex
Choledochoscope diameter	2.4 mm	3.5 mm
Pixels	16*10^4^	12*10^4^
Depth of field	0.3–20 mm	Low
Frond-end direction adjustment	Guidewire adjustment	Four-quadrant adjustment
Front-end flexibility	Good	Hard
Tilting up angle	>90°	30–40°
Water injection rate	0.8 mL/s	0.7 mL/s
Water injection distance	10.0 cm	7.0 cm
Cost	Low	High
Categorization	Accessory/consumable	Choledochoscope

## Discussion

The current choledochoscope products on the market have their respective features and also shortcomings. Our study intended to use miniature photography technology to innovatively develop an ERCP technique-specific superfine high-resolution catheter system in the digestive endoscopy field for visual examination of bile and pancreatic ducts. This system is inserted via the duodenoscope biopsy channel to enter the gallbladder and intrahepatic bile ducts for observation and is a catheter accessory guided by the guidewire to facilitate clinical use.

The SpyGlass™ developed by Boston Scientific is full-featured, with the front end adjustable from multiple angles to enable direct-vision biopsy or bile duct stone lithotripsy (14). However, the outer diameter (3.5 mm) of its front end cannot be further reduced because its light source is transmitted to the front end of the camera via an optical fiber. Multiple clinical needs are taken into account and multiple powerful features, such as a continuous injection pump, supporting superfine biopsy forceps, and four-quadrant adjustable steering wire, are integrated. SpyGlass™ thus cannot enter the gallbladder or intrahepatic third-order and fourth-order bile ducts in clinical practice. Furthermore, as the SpyGlass™ DS has a thick front end that contains complex components, the front end is hard and not very flexible. As a result, it requires a big incision or dilatation of the duodenal papilla for insertion during the ERCP, and during the insertion process, the forceps lifter requires great effort to be lifted. Moreover, the optical fiber used by the SpyGlass™ DS can only be bent to a limited extent, and when squeezed, the fiber will become attenuated and affect the imaging, causing fiber damage in cases of any carelessness in the operation process (15).

The disposable ERCP and photography catheter system was designed and developed by us to solve practical problems in clinical practice and meet clinical needs, with features of low cost and single use as an accessory/consumable. The ability of four-quadrant manipulation was given up because the integrated catheter design of the disposable superfine catheter for visual examination of bile and pancreatic ducts enables the *in vitro* control of the handle to achieve 360° of accurate rotation of the photography catheter so as to effectively adjust the advancing direction of the photography front end. The photography front end can only be bent to a limited extent in the bile ducts with a relative diameter of about 8 mm or less, and inside the narrow space of small-diameter intrahepatic bile ducts, close observation can be achieved without the need for large-angle adjustment. Guided by the superfine guidewire, the disposable superfine catheter for visual examination of bile and pancreatic ducts can enter different bile ducts in reliance on its advantage of a fine outer diameter. Although its overall diameter is only 2.4 mm, it is still superior to SpyGlass™ DS in terms of water injection rate. Furthermore, the 0.16 megapixel camera with a resolution of 400 × 400 ensures the ability to observe the bile duct wall mucosa and blood vessels.

The disposable superfine catheter for visual examination of bile and pancreatic ducts currently does not have direct-vision biopsy ability. We mainly conducted the autopsy by marking the location of the catheter under X-ray and, after withdrawing the catheter, inserting the transnasal gastroscope to use the biopsy forceps. The indirect biopsy method has the disadvantage of repeated changes of accessories; however, it can largely reduce the medical cost.

In conclusion, the focus of clinical medical treatment has gradually shifted toward the microscopic, visual, precise, and super minimally invasive aspects with the rapid development of science and technology and the continuous improvement of the manufacturing level. The fast development of the indirect per-oral visual choledochoscopes allows endoscopist to safely and efficiently “enter” the biliary system to perform diagnosis and treatment and has greatly expanded the clinical indicators of biliary endoscopy. The combination of new materials and imaging technology with special accessories designed and developed by us is expected to address the above aspects and make biliary disease diagnosis and treatment more efficient, convenient, and economical.

## Data Availability

The original contributions presented in the study are included in the article/Supplementary Material, further inquiries can be directed to the corresponding author/s.
